# Prevention of Bone Loss in a Model of Postmenopausal Osteoporosis through Adrenomedullin Inhibition

**DOI:** 10.3389/fphys.2016.00280

**Published:** 2016-06-30

**Authors:** Sonia Martínez-Herrero, Ignacio M. Larrayoz, Laura Ochoa-Callejero, Luis J. Fernández, Alexis Allueva, Ignacio Ochoa, Alfredo Martínez

**Affiliations:** ^1^Angiogenesis Interest Group, Oncology Area, Center for Biomedical Research of La Rioja, Fundación Rioja SaludLogroño, Spain; ^2^Centro de Investigación Biomédica en Red, Aragon Institute of Health SciencesZaragoza, Spain; ^3^Group of Structural Mechanics and Materials Modelling, Aragón Institute of Engineering Research (I3A), University of ZaragozaZaragoza, Spain

**Keywords:** adrenomedullin, inducible knockout, bone physiology, ovariectomized mice, osteoporosis, small molecule inhibitor

## Abstract

Despite recent advances in the understanding and treatment options for osteoporosis, this condition remains a serious public health issue. Adrenomedullin (AM) is a regulatory peptide with reported activity on bone remodeling. To better understand this relationship we built an inducible knockout for AM. An outstanding feature of knockout mice is their heavier weight due, in part, to the presence of denser bones. The femur of knockout animals was denser, had more trabeculae, and a thicker growth plate than wild type littermates. The endocrine influence of AM on bone seems to be elicited through an indirect mechanism involving, at least, the regulation of insulin, glucose, ghrelin, and calcitonin gene-related peptide (CGRP). To confirm the data we performed a pharmacological approach using the AM inhibitor 16311 in a mouse model of osteoporosis. Ovariectomized females showed significant bone mass loss, whereas ovariectomized females treated with 16311 had similar bone density to sham operated females. In conclusion, we propose the use of AM inhibitors for the treatment of osteoporosis and other conditions leading to the loss of bone mass.

## Introduction

Osteoporosis is a serious public health issue, especially taking into consideration the growing aging population in developed countries. Osteoporosis is characterized by a reduction in bones mineral density and a deterioration of bone microarchitecture, which results in a higher risk of bone fractures (NIH Consensus Development Panel on Osteoporosis Prevention, Diagnosis, and Therapy, 2001; Sambrook and Cooper, 2006). Osteoporosis is more frequent in women, especially after menopause. Estrogen deficiency increases bone resorption, leading to a net loss of bone mass by unbalanced bone deposition by osteoblasts and bone resorption by osteoclasts (Andreopoulou and Bockman, 2015). Nowadays, two major classes of treatments are applied to osteoporosis patients: (i) antiresorptive medications which prevent bone loss by blocking osteoclast formation, survival or activity, such as bisphosphonates, the RANKL-neutralizing monoclonal antibody, denosumab, and cathepsin K inhibitors, such as odanacatib; and (ii) anabolic agents which stimulate osteoblasts, such as PTH1-34 (teriparatide), which is currently approved as a daily injection, and other analogs of PTH and PTHrP which are in development (Makras et al., 2015). In any case, osteoporosis patients could benefit from the generation of new drugs that exploit novel biological pathways, yet unexplored.

Adrenomedullin (AM) is a 52 amino acid peptide with a ubiquitous distribution and many physiological functions (Lopez and Martinez, 2002), one of which is the regulation of insulin secretion (Martinez et al., 1996). AM is coded by the *Adm* gene located in mouse chromosome 7. The AM receptor consists on a 7-transmembrane domain protein called calcitonin receptor-like receptor (CLR) in combination with a single transmembrane domain protein known as receptor activity modifying protein (RAMP) (McLatchie et al., 1998). Using immunohistochemical techniques, it has been shown that AM is expressed in the developing chick limb buds (Seghatoleslami et al., 2002) and in chondrocytes and osteoblasts in the bone's growth plate (Montuenga et al., 1997), suggesting a potential role in bone development. It has also been shown that AM binds to osteoblasts (Naot et al., 2001), inducing the growth of both osteoblasts (Cornish et al., 1997) and chondrocytes (Cornish et al., 2003) *in vitro*. In addition, AM reduces apoptotic cell death in serum-starved osteoblasts (Uzan et al., 2008). All these actions result in a net bone mass increase in *ex vivo* experiments (Cornish et al., 1997; Siclari et al., 2014). To carry out formal studies on the correlation between AM and bone development *in vivo*, a knockout model for either AM or its receptors is needed. Results from several groups have shown that early abrogation of the gene coding for AM or some of its receptor components results in 100% embryo lethality due to serious vascular abnormalities (Caron and Smithies, 2001; Shindo et al., 2001; Dackor et al., 2006). To circumvent this problem, we developed a conditional knockout for AM using *Cre/loxP* technology and have shown that it works well when targeting specific cell types such as neurons (Fernandez et al., 2008), endothelial cells (Koyama et al., 2013), or club cells in the lung (Garcia-Sanmartin et al., 2016). Here we describe the methodology to generate an inducible model in which the AM gene can be eliminated in adult mice. These animals survive the abrogation of the gene and constitute a good model to study the physiological implications of AM, including its impact on bone biology.

Several pharmacological interventions have been described to reduce the physiological activity of AM. These include a monoclonal antibody (Martinez et al., 1996), polyclonal antibodies against either the peptide (Martinez et al., 1995; Ouafik et al., 2002) or the receptors (Kaafarani et al., 2009), the peptide fragment AM_22−52_ (Ishikawa et al., 2003), and small interfering RNAs (Ramachandran et al., 2007). In addition, several small molecules have been identified which can either increase or decrease AM functions (Martinez et al., 2004a; Roldos et al., 2008). One of these, the inhibitory molecule 16311, has been used in this study to demonstrate pharmacologically the effects of AM inhibition in a mouse model of osteoporosis.

## Materials and methods

### Generation of inducible knockout mice

Mice where the *Adm* gene was surrounded by *loxP* sequences (“floxed”) were generated in our lab and previously characterized (Fernandez et al., 2008). These animals were crossed with transgenic mice expressing Cre recombinase under the control of a tetracycline-responsive promoter element (tetO) (Strain Number 6234, The Jackson Laboratory, Bar Arbor, ME) and with mutant mice having widespread expression of an optimized form of reverse tetracycline-controlled transactivator (rtTA-M_2_) protein (Strain Number 6965, The Jackson Laboratory). All three strains had been previously backcrossed to a C57BL/6 genetic background for several generations. Triple transgenic animals are viable and lead a normal life. For experiments, the following two genotypes were selected: normal controls (homozygous for the *Adm* wild type allele, tetO-Cre, and rtTA) and knockout animals (homozygous for the “floxed” *Adm* allele, tetO-Cre, and rtTA).

All procedures involving animals were carried out in accordance with the European Communities Council Directive (2010/63/UE) and Spanish legislation (RD53/2013) on animal experiments and with approval from the ethical committee on animal welfare of our institution (Órgano Encargado del Bienestar Animal del Centro de Investigación Biomédica de La Rioja, OEBA-CIBIR). Every week, animals were weighed and inspected to ensure a healthy status. Humane endpoints were defined (piloerection, lack of movement, protective posture, significant weight loss) and animals expressing those symptoms were euthanized. A symptom-free survival curve was built using those data. For euthanasia, animals were intraperitoneally injected with a lethal dose of anesthetic: 300 mg/Kg ketamine (Imalgene, Merial Laboratorios, Barcelona, Spain) + 30 mg/Kg xylazine (Xilagesic, Proyma Ganadera, Ciudad Real, Spain). After organ collection, mice were decapitated to ensure death.

### Deletion induction and molecular comprobation

Male and female 9 week-old mice of both genotypes (control and “floxed” mice) were exposed to 2 mg/ml doxycycline in the drinking water, supplemented with 5% sucrose, for 15 days. After this period, mice were provided with regular water and allowed to rest for at least 4 weeks before performing any experiments. At this point, a group of animals (*n* = 5 per sex and genotype) were sacrificed and several organs collected and frozen in liquid nitrogen. DNA was extracted and subjected to PCR with primers located outside the *loxP* sequences: P1 (sense): AAGGGAAGTCCTGCTCCAGT, and P2 (antisense): GCCTTAGCTCAGGTCCAGTG. The expected amplicon size is 2500 bp for the wild type allele and 600 bp after the *Adm* gene has been eliminated.

### RIA and ELISA blood protein determination

At the time of sacrifice, blood (*n* = 40) was collected from the heart and serum was prepared and frozen until further analysis. The concentrations of AM were determined using a commercially available RIA kit (Phoenix Pharmaceuticals, Inc., Karlsruhe, Germany). Samples (1 ml) were initially diluted in an equal volume of 0.1% alkali-treated casein in PBS, pH 7.4, and applied to pre-washed reverse-phase Sep-Pak C-18 cartridges (Waters Corp., Milford, MA). The peptide fraction was eluted from the C18 matrix with 3 ml 80% isopropanol containing 0.125N HCl and freeze-dried overnight, as described (Martinez et al., 1999). AM levels found in lyophilized extracts were then determined by RIA following manufacturer's instructions. Commercially available ELISA kits for acetylated ghrelin (Millipore Corporation, St. Charles, MO) and CGRP (USCN Life Sciences, Beijing, China) were purchased and peptide levels were quantified following manufacturer's instructions.

### RNA isolation and quantitative real time PCR (qRT-PCR)

Tissues were homogenized with TRIzol (Invitrogen, Madrid, Spain) and RNA was isolated with RNeasy Mini kit (Qiagen, Germantown, MD). Three micrograms of total RNA were treated with 0.5 μl DNAseI (Invitrogen) and reverse-transcribed into first-strand cDNA using random primers and the SuperScript III kit (Invitrogen). Reverse transcriptase was omitted in control reactions, where the absence of PCR-amplified DNA confirmed lack of contamination from genomic DNA. Resulting cDNA was mixed with SYBR Green PCR Master Mix (Invitrogen) for quantitative real time polymerase chain reaction (qRT-PCR) using 0.3 μM forward and reverse oligonucleotide primers (AMf: ATT GAA CAG TCG GGC GAG TA; AMr: CTT GGT CTT GGG TTC CTC TG; GAPDHf: CAT GTT CCA GTA TGA CTC CAC TC; GAPDHr: GGC CTC ACC CCA TTT GAT GT). Quantitative measures were performed using a 7300 Real Time PCR System (Applied Biosystems, Carlsbad, CA). Cycling conditions were an initial denaturation at 95°C for 10 min, followed by 40 cycles of 95°C for 15 s and 60°C for 1 min. At the end, a dissociation curve was implemented from 60 to 95°C to validate amplicon specificity. Gene expression was calculated using absolute quantification by interpolation into a standard curve. All values were divided by the expression of the house keeping gene GAPDH.

### Blood pressure measurements

Blood pressure was measured in conscious animals (*n* = 32) by the tail-cuff method (CODA, Kent Scientific, Torrington, CT). Briefly, each animal was acclimated for at least 10 practice sessions for a minimum of 15 min, then blood pressure parameters were recorded during 5 consecutive days. In each recording session, 1 set of 5 acclimation cycles followed by 3 sets of 10 acquisition cycles were performed, and the average of the last 20 successful recordings was used for calculating systolic and diastolic blood pressure, heart rate, tail blood flow, and tail blood volume.

### Glucose tolerance tests

Mice (*n* = 40) were fasted for 6 h and blood samples were collected from the tail vein. Glucose levels were measured with a clinical monitor (Contour XT, Bayer, Madrid, Spain) to establish a baseline glucose level (T0). They were then injected intraperitoneally with 2.0 g glucose/kg body weight. Blood samples were taken at 15, 30, 60, and 120 min after injection and glucose measured as before.

### Bone imaging, histology, and immunohistochemistry

At the time of sacrifice (*n* = 40), several tissues including femur, pancreas, stomach, liver, and abdominal fat, were weighed and fixed in 10% buffered formalin. Microtomography of the femurs was carried out in a blinded fashion to define the bone volume, as well as the bone density among the different groups. The image files (DICOM- Digital Imaging and Communication in Medicine) provided by the microCT were the main input for building the geometric model of the femurs. Images of the mouse legs were obtained by a rotational scanning of 360°. A GE Healthcare eXplore locus SP microCT was used, with an x-ray filter number 3, 75 kV voltage, and 90 mA power. The resolution of the equipment was 34 μm. Six hundred DICOM files were obtained for each sample (one image for each rotation of 1.06°). The quality of the final model and its similarity with the original sample are directly related to the degree of resolution and the number of segmented images. The volume segmentation was made by sweeping all the scanned slides. A phantom with known density was used to normalize values. Bone mass was expressed either as Hounsfield units or as the bone volume to total volume fraction (BV/TV) (Jeyabalan et al., 2013; Kang et al., 2014). After the images had been taken, bone samples were decalcified with Biodec R (Bio-Optica, Milano, Italy), dehydrated, and embedded in paraffin. All other tissues were paraffin-embedded without decalcification. Tissue sections (3-μm thick) were stained with hematoxylin-eosin and Masson's trichrome. The proportion trabecular to laminar bone was calculated with image processing software (ImageJ) and growth plate thickness was measured in all specimens.

In addition, fragments of duodenum from both genotypes were fixed in 10% buffered formalin, paraffin embedded, and sections were immunohistochemically stained for Lyve1. Internal peroxidase activity was blocked by incubation with H_2_O_2_ in methanol and antigen retrieval was accomplished in 10 mM sodium citrate, 0.5% Tween 20, pH 6.0, for 20 min at 95°C. Non-specific binding was blocked with normal goat serum. A rabbit anti-Lyve1 antibody (Abcam, Cambridge, UK) was applied at a concentration 1:1000 at 4°C overnight. A biotinylated goat anti-rabbit immunoglobulin (1:200), the Vectastain Elite ABC kit (Vector Laboratories, Burlingame, CA), and diaminobenzydine (Sigma-Aldrich, Madrid, Spain) were used to detect the primary antibody. At the end, sections were lightly counterstained with hematoxylin.

### Osteoporosis mouse model

Forty 8-week old female C57BL/6 mice (Charles River, Barcelona, Spain) were used for this experiment. Twenty of them were ovariectomized and the other 20 were sham operated. On each group, half of the mice (*n* = 10) were injected i.p. 3 times a week with PBS as a vehicle, whereas the other 10 mice received 20 nmols/kg of small molecule 16311 dissolved in PBS. After 5 weeks of treatment, mice were deeply anesthesized, blood was extracted from the heart to study toxicity markers, and the femurs were fixed in 10% buffered formalin. Bone density was analyzed by micro-CT as above. Small molecule 16311 was custom made by Centro de Química Aplicada y Biotecnología (University of Alcalá, Madrid, Spain). Concentration and dosing regime was chosen based on previous studies with blood pressure regulation (Martinez et al., 2004a).

### Statistics

Symptom-free survival data at 100 weeks of age were displayed as Kaplan-Meier curves and analyzed with Log-rank Mantel-Cox statistics. Blood pressure parameters were analyzed by Student's *t*-test after confirming data normalcy and homoscedasticity. Glucose tolerance tests, weight patterns, and osteoporosis treatments were studied with 2-way ANOVA. Kruskal-Wallis followed by Dunn's *post-hoc* test was used for all other data. All studies were performed with GraphPad Prism version 5.02.

## Results

### Characterization of the inducible knockout

**Figure 1:**
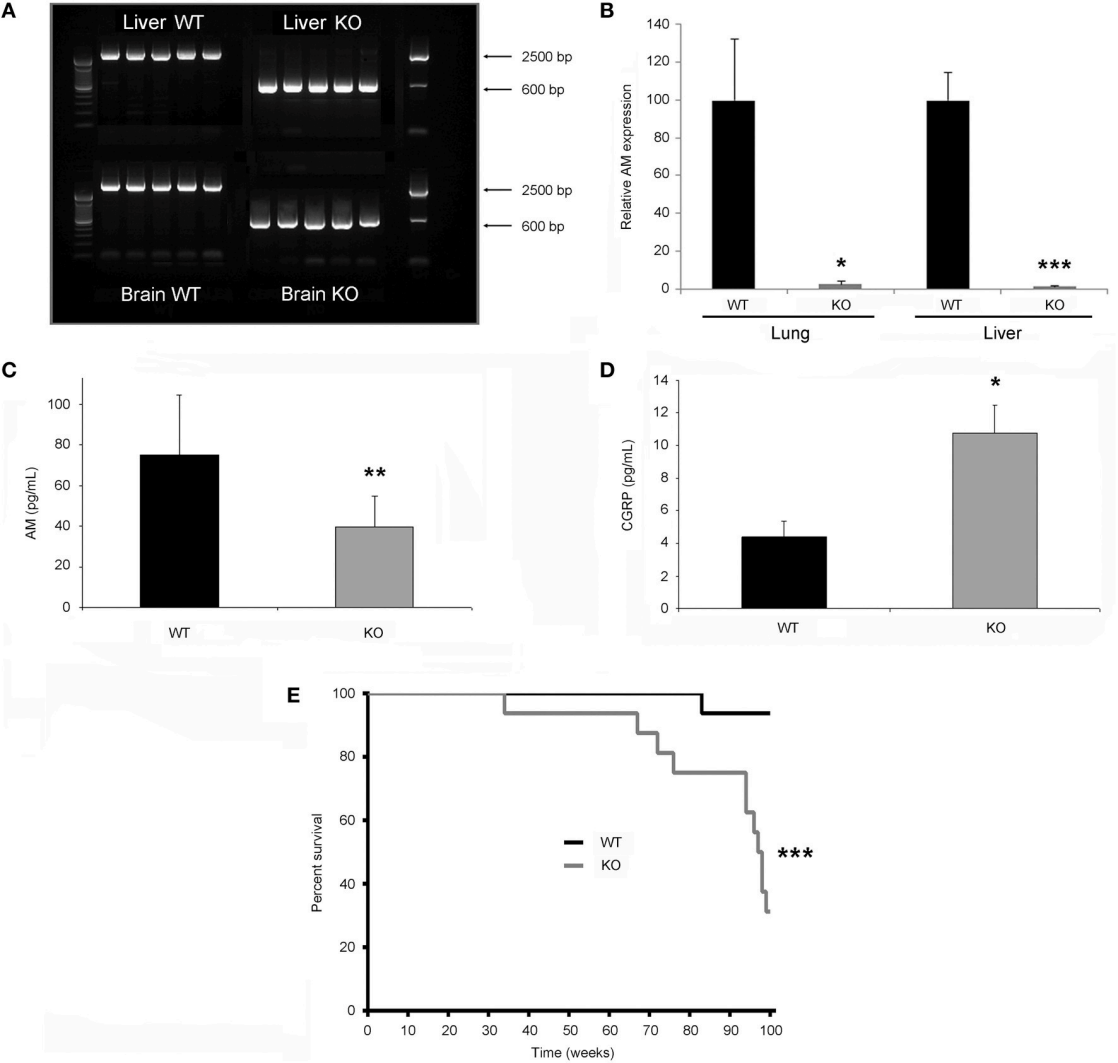
**Characterization of the knockout phenotype**. **(A)** Liver and brain fresh tissue was collected from 5 wild type (WT) and 5 knockout (KO) male mice and subjected to PCR with primers which distinguish the unmodified *Adm* gene (~2500 bp) from the recombined allele (~600 bp). **(B)** mRNA expression for AM was determined in the lung and liver. Values represent a relative AM/GAPDH ratio (*n* = 6 per group, all males). **(C)** A radioimmunoassay for AM was performed in blood serum from WT and KO animals (*n* = 12 per group, all males). **(D)** CGRP serum contents were calculated by ELISA (*n* = 6 per group, all males). **(E)** Mouse symptom-free survival (*n* = 32, 16 males, 16 females) was followed up to 100 weeks of age and is represented by Kaplan-Meier curves. Statistically significant differences between genotypes are expressed by asterisks. ^*^p < 0.05; ^**^p < 0.01; ^***^p < 0.001.

Mice with the proper genotype were obtained and subjected to doxycycline treatment for 2 weeks. Confirmation of *Adm* deletion was performed by PCR in numerous organs (brain, pituitary, lung, heart, liver, pancreas, small intestine, colon, gonads, kidney, skeletal muscle, and several others). In all the WT animals a band of around 2500 bp was obtained, indicating the presence of the gene despite the treatment with doxycycline. In contrast, all tissues obtained from the KO mice presented a single band of ~600 bp, showing a complete deletion of *Adm* (Figure 1A). To test whether this gene targeting resulted in expression reduction, quantitative real time PCR was performed in several organs, including the lung and liver, 6 weeks after initiating doxycycline treatment, and a marked reduction in AM mRNA was achieved (Figure 1B). Furthermore, levels of AM were measured in blood serum. KO animals had significantly lower levels of AM than their WT littermates (Figure 1C). Calcitonin gene-related peptide (CGRP) belongs to the same peptide family as AM and has been shown to increase when AM levels are reduced (Fernandez et al., 2010). We checked the levels of this peptide in the animals lacking AM by ELISA and we found a significant increase when compared with WT animals (Figure 1D). Symptom-free survival of the gene-targeted mice was followed up to 100 weeks. KO mice had a significantly lower survival than their WT littermates (Figure 1E). Survival began to differ between genotypes at 94 weeks, providing a long time to perform physiological experiments in *Adm*-null mice. This observation is in sharp contrast with what happens in fetal mice where lack of this gene results in embryo lethality (Caron and Smithies, 2001). The more profusely studied physiological action of AM is blood pressure regulation (Lopez and Martinez, 2002), so we tested how the total elimination of *Adm* influenced this parameter. There was a significant decrease in diastolic blood pressure in the KOs whereas there was no change in systolic blood pressure. In addition, KO animals had a higher pulse frequency and lower blood flow rate and tail blood volume than their WT counterparts (Table 1).

**Table 1:** **Blood pressure parameters as measured with the CODA system in wild type (WT) and knockout (KO) mice (***n*** = 16 per group)**.

	**WT**	**KO**	***p***	
Systolic blood pressure (mm Hg)	138.7 ± 1.15	140.0 ± 0.99	0.37	n.s.
Diastolic blood pressure (mm Hg)	108.5 ± 1.00	103.3 ± 0.95	0.0002	^***^
Pulse (beats/min)	705.1 ± 3.18	719.2 ± 3.74	0.0042	^**^
Blood flow rate (mL/min)	15.9 ± 0.34	11.8 ± 0.25	< 0.0001	^***^
Tail blood volume (μL)	29.6 ± 0.66	26.3 ± 0.48	< 0.0001	^***^

*Values are mean ± SEM of all valid measurements. Statistical significance was calculated with Student's t test*.

** p < 0.01;

*** *p < 0.001*.

### Knockout *Adm* mice gain weight rapidly

**Figure 2:**
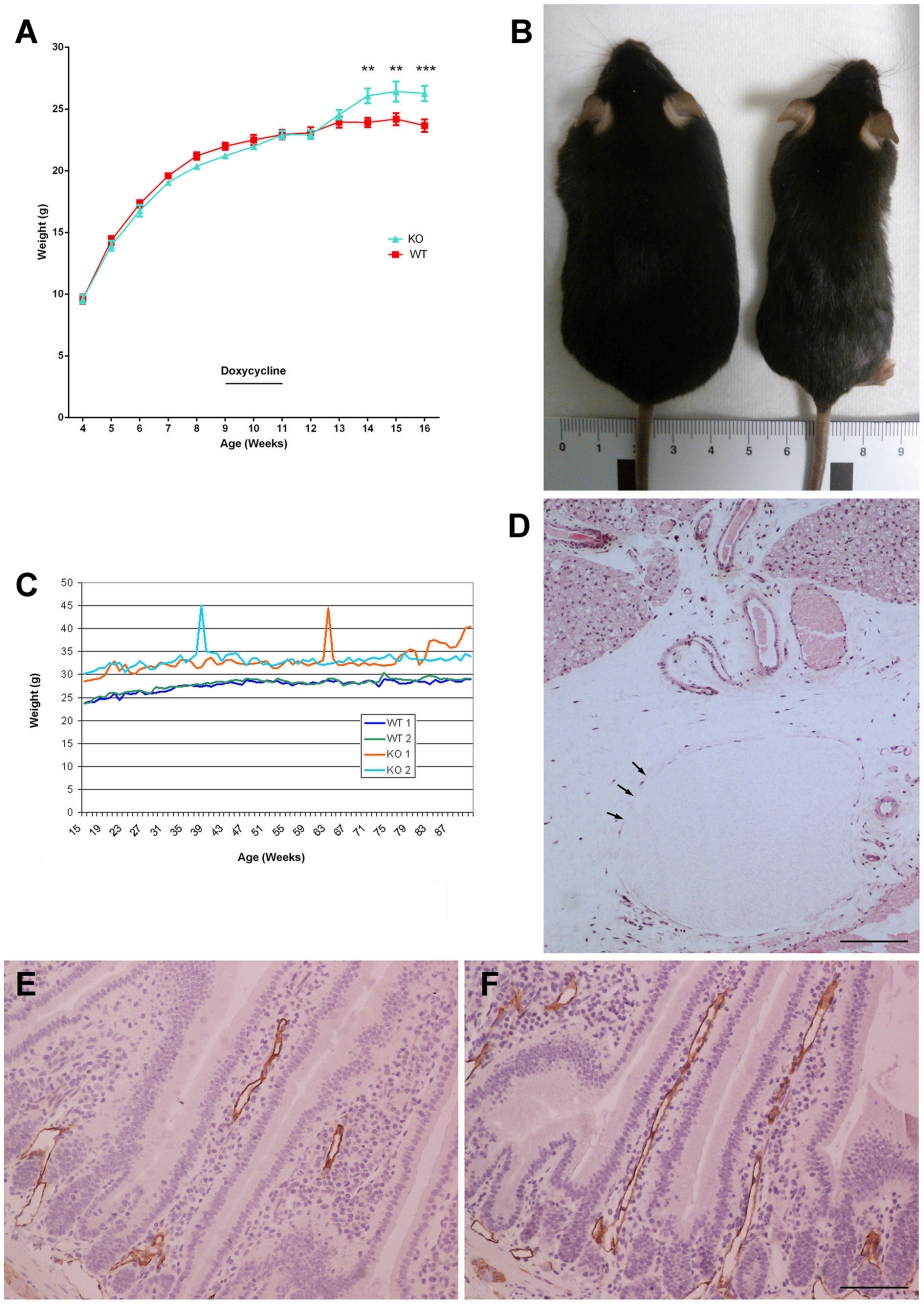
**Weight differences between genotypes**. Mouse weight was measured weekly from weaning (at 4 weeks of age) in males for both wild type (WT) and knockout (KO) mice. **(A)** Following doxycycline treatment (weeks 9–11) and subsequent gene recombination, the mean weight of the KO mice became significantly higher than that of their WT littermates. Each point represents the mean ± SD of all mice (*n* = 10 per group). ^**^p < 0.01; ^***^p < 0.001. **(B)** Sporadically, individual KO mice (left) gained weight very rapidly, becoming much larger than the WT controls (right). **(C)** Interestingly, following the weight of individual mice through time, these large animals were able to lose this accumulated weight very rapidly, as well, returning to the mean weight of their genotype within a week. **(D)** Histological analysis of the tissues of an animal at the peak of its weight gain shows a widespread interstitial edema and lymphatic vessel occlusion (arrows). The image shows a section of the neck area, close to a salivary gland, stained with hematoxylin-eosin. Bar = 100 μm. Cross-sections of duodenum in WT **(E)** and KO **(F)** mice stained with an antibody against Lyve1 (brown color) and counterstained with hematoxylin. Bar = 50 μm.

We observed that the population of KO mice gained weight rapidly after exposure to doxycycline and consequent induction of gene deletion (Figure 2A), with a mean overweight of 4.5 g per animal. We tried to identify the cause for these changes and different organs were studied. The weight of several organs is shown in Table 2. No significant differences were found between genotypes. The only difference we found was the presence of denser bones in the KOs (see below). Interestingly, some individual mice (about 12% of the male KO animals) gained a lot of weight in short periods of time, being able to lose it again after a few days (Figures 2B,C). At the peak of their weight gain, these animals suffered from transitory generalized interstitial edema, a condition also known as anasarca. At this time, histological observation showed that the connective tissue was swamped by interstitial fluid and the lymphatic vessels were largely distended (Figure 2D). To check whether this lymphedema was present in KO animals not affected by anasarca, we studied the morphology of lymphatic vessels in the intestine staining them with the specific marker Lyve1, and no major differences were observed (Figures 2E,F).

**Table 2:** **Relative weight, expressed as percentage of total body weight, of several organs in wild type (WT), and knockout (KO) male mice**.

	**WT**	**KO**	***p***
Abdominal fat	0.68 ± 0.08	0.79 ± 0.05	0.13
Pancreas	0.94 ± 0.22	0.81 ± 0.19	0.16
Stomach	0.56 ± 0.16	0.62 ± 0.06	0.25
Liver	4.62 ± 0.46	5.05 ± 0.52	0.06

*None of the differences reached statistical significance*.

### KO mice present denser bones

**Figure 3:**
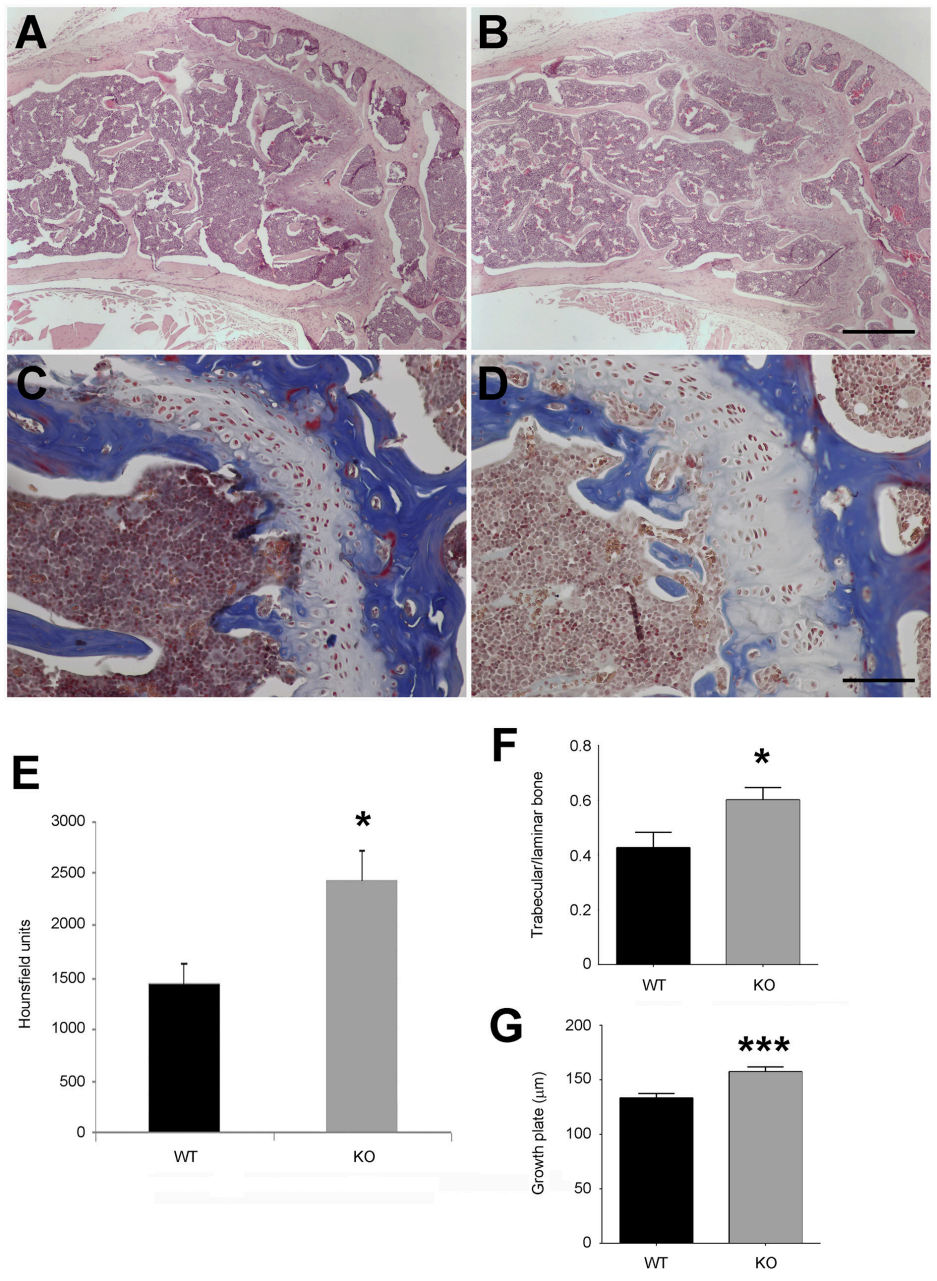
**Bone parameters in WT and KO mice**. Histological sections of the head of the femur in WT **(A,C)** and KO **(B,D)** animals, stained with hematoxylin and eosin **(A,B)** or with Masson's trichrome **(C,D)**. Femur bone density was measured in both genotypes **(E)**. The ratio trabecular vs. laminar bone **(F)** and the thickness of the growth plate **(G)** was calculated for both genotypes. Each bar represents the mean ± SD of all mice. ^*^p < 0.05; ^***^p < 0.001. Bar for **(A,B)** = 500 μm. Bar for **(C,D)** = 100 μm.

Histology of the femur showed that the relative surface occupied by trabeculae was higher in KO animals than in their WT counterparts (Figures 3A,B,F). In addition, the growth plate at the epiphysis was thicker in the animals lacking AM (Figures 3C,D,G). Furthermore, micro-CT measurements of the femur also showed a higher bone density in the KOs (Figure 3E), with no changes in bone length.

### Knockout mice gain bone mass through an indirect mechanism

**Figure 4:**
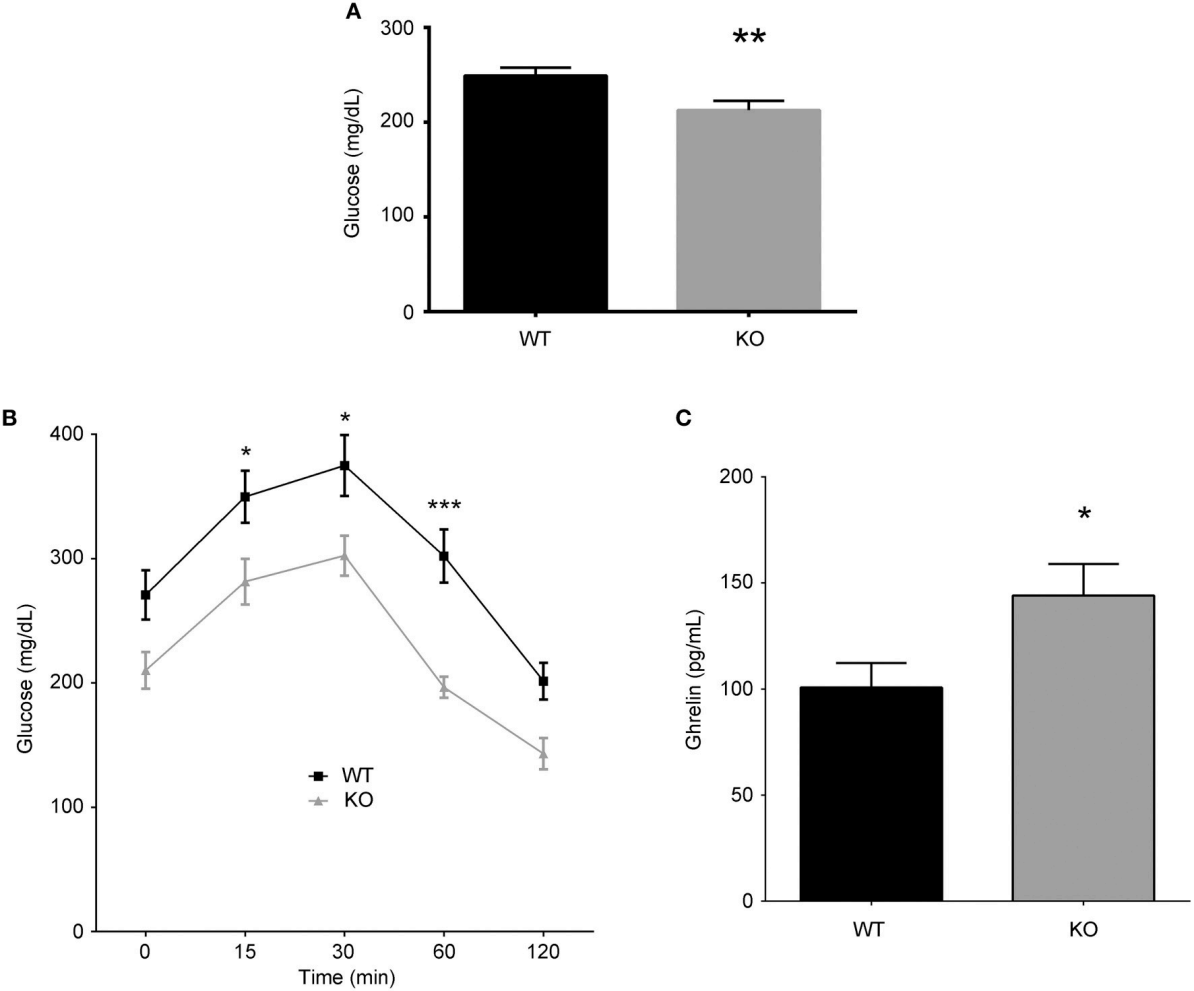
**Potential indirect mechanism of AM-dependent bone density regulation**. **(A)** Basal fasting glucose levels were significantly lower in KO animals than in their WT counterparts (*n* = 30 per group). **(B)** Following a glucose tolerance test, significant differences were observed in the blood glucose levels comparing both genotypes, with the KO mice having lower values than the WT controls (*n* = 10 per group). **(C)** Acetylated ghrelin levels were measured in the blood of both genotypes. In this case, the levels of active ghrelin were significantly higher in the KO animals (*n* = 10 per group). Each point represents the mean ± SD of all mice. ^*^p < 0.05; ^**^p < 0.01; ^***^p < 0.001.

Since previous *in vitro* reports indicated that AM increases bone mass (Cornish et al., 1997, 2003; Naot et al., 2001; Uzan et al., 2008), we would have expected a lower bone mass in animals lacking *Adm*. But, since we obtained the reverse result (Figure 3), we hypothesized that our observations must be due to an indirect systemic effect of AM. A possible mechanism may involve the increase in CGRP shown in Figure 1D, since CGRP can induce bone growth through stimulation of bone mesenchymal stem cells (Liang et al., 2015). Also, AM has been shown to reduce insulin secretion in the pancreas, thus increasing glucose levels (Martinez et al., 1996). We tested whether glycemia was affected in the KOs and found that animals lacking AM had lower fasting basal glucose levels than their WT littermates (Figure 4A). These differences in glycemia were also maintained through a glucose tolerance test (Figure 4B). It has been shown that low glucose induces ghrelin secretion from the stomach (Goldstein et al., 2011). Accordingly, ghrelin levels in the blood of our KOs are significantly higher than in the WTs as tested by ELISA (Figure 4C). Closing the circle, the literature tells us that ghrelin promotes bone formation either through the induction of growth hormone or through growth hormone-independent mechanisms (Tong et al., 2012; Delhanty et al., 2014b; Wee and Baldock, 2014).

### An AM inhibitor prevents osteoporosis

**Figure 5:**
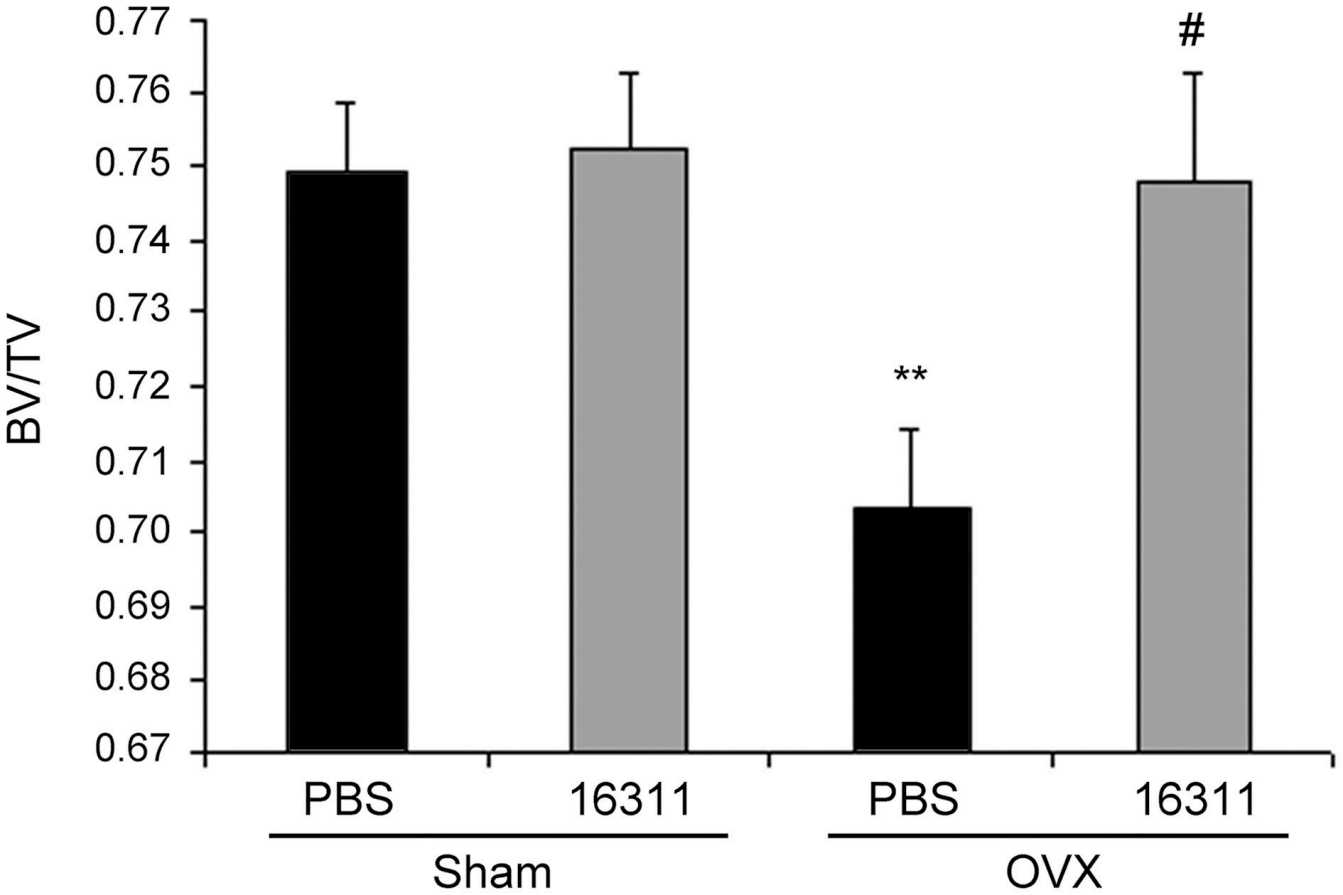
**Small molecule 16311 prevents bone loss**. Female mice were ovariectomized (OVX) or Sham operated (Sham) and then treated for 5 weeks with PBS (as a vehicle) or with 20 nmols/kg of small molecule 16311 dissolved in PBS, and the bone density of their femurs was measured by microCT (expressed as BV/TV) (*n* = 10 female mice/group). As expected, untreated ovariectomized mice suffered a significant bone loss when compared to Sham females (^**^p < 0.01). On the other hand, ovariectomized females treated with 16311 had undistinguishable density from Sham controls (*p*>0.05) and significantly more bone density than untreated ovariectomized females (^#^*p* < 0.05). Each bar represents the mean ± SD of all mice (*n* = 10 per group).

**Figure 6:**
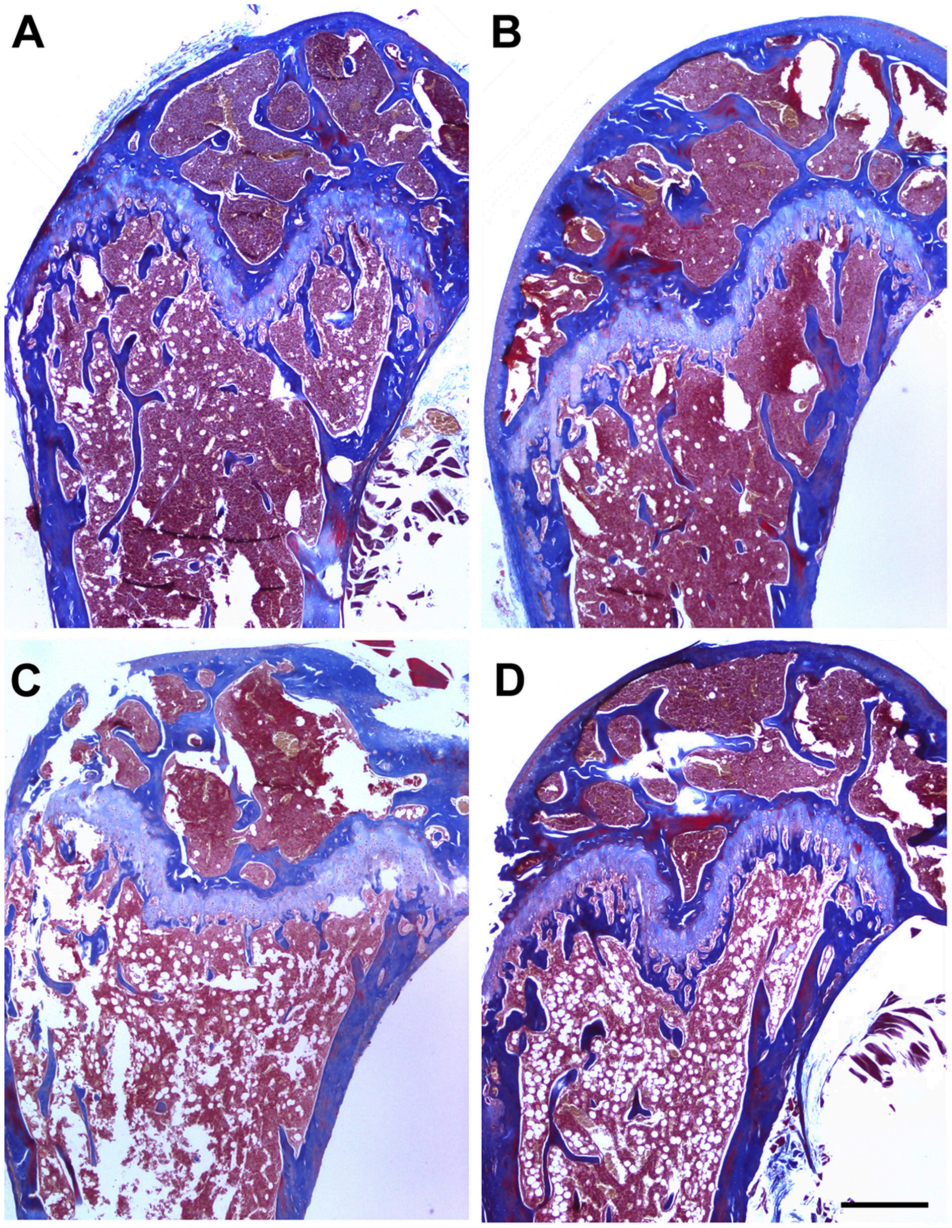
**Small molecule 16311 prevents loss of trabeculae**. Representative histological sections of the head of the femur of animals in the ovarectomy experiment stained with Masson's trichrome. Photographs represent sham-operated animals treated with PBS **(A)** or with the small molecule **(B)** and ovarectomized females treated with PBS **(C)** or with the small molecule **(D)**. Bar = 500 μm.

If our hypothesis expressed above is correct, any effective inhibitor of AM should increase bone mass or prevent its loss in an *in vivo* setting. To test this, we used an osteoporosis mouse model where ovariectomy produces estrogen deficiency and bone loss. Small molecule 16311 was used as an AM inhibitor (Martinez et al., 2004a). As expected, untreated ovariectomized females suffered significant bone loss when compared with fertile females (Figure 5). Interestingly, treatment with 16311 had no effect on normal females, but completely prevented bone loss in ovariectomized mice (Figure 5). This was also confirmed by histological observation of the bones (Figure 6). At the end of the treatment, blood was extracted and toxicity markers were studied. Treatment with small molecule 16311 did not induce any blood, liver, or renal toxicity at the concentrations and periodicity used in this experiment (Table 3).

**Table 3:** **Toxicity analysis in the blood of ovariectomized (OVX) mice treated and untreated with 16311**.

		**OVX + PBS**	**OVX + 16311**
Biochemistry	Urea (mg/dL)	18.21 ± 3.33	18.50 ± 3.78
	Creatinine (mg/dL)	0.13 ± 0.01	0.13 ± 0.02
	ALT (IU/L)	39.52 ± 2.33	42.80 ± 7.08
	AST (IU/L)	92.00 ± 5.62	82.20 ± 7.75
	ALKP (IU/L)	40.03 ± 34.27	43.60 ± 35.63
	GGT (IU/L)	1.14 ± 0.58	0.56 ± 0.36
Hemogram	MCV (%)	31.40 ± 0.33	30.58 ± 0.31
	RBC (10^6^/μL)	7.66 ± 0.08	7.46 ± 0.09
	Hb (g/dL)	12.18 ± 0.15	11.78 ± 0.17
	WBC (10^3^/μL)	6.10 ± 0.64	4.96 ± 0.68

*Values are the mean ± SEM of all animals in the group (n = 10 per group). All numbers are within normal values for C57BL/6 mice*.

## Discussion

In this study we have shown that elimination of the gene coding for AM in adult mice is compatible with life. AM deficiency results in an increased weight of the animals which is accompanied by an increase in bone density, at least in the femur. This increased bone mass may be achieved through an indirect mechanism involving regulation of glycemia and ghrelin and CGRP secretion. The hypothesis that AM inhibition could prevent bone mass loss was demonstrated with a small molecule inhibitor of AM in a mouse model of osteoporosis.

The choice of proper controls is an important step in any experiment, but especially so when working with knockout models. We had several theoretical options. One of them was to compare mice with the same genotype (homozygous for the “floxed” allele, tetO-Cre, and rtTA) that were treated (KO) or untreated (WT) with doxycycline. Recent studies have shown that doxycycline is not just an antibiotic that kills bacteria and has no influence in eukaryotic cells; on the contrary this tetracycline modifies many cellular pathways in vertebrates (Larrayoz et al., 2013), and therefore sharp differences between treated and untreated animals may occur, independently of their genotype. In addition, there is always the possibility of some leakage in the control of the promoter, resulting in “WT” animals with partial deletion of the gene (Backman et al., 2009). In consequence, we decided to treat both the KO and the WT animals with the same dose of doxycycline, choosing different genotypes that respond (KO) or not (WT) to this treatment.

When looking at the circulating levels of AM in KO mice we found a significant decrease of AM but these levels were higher than expected, despite the AM mRNA expression being close to zero. These results may be explained in two ways. First, we may hypothesize the existence of a reservoir that keeps providing AM even after the gene is eliminated. In fact, the existence of an AM serum binding protein, complement factor H, was described (Pio et al., 2001) and this protein is able to protect AM from MMP2-mediated degradation (Martinez et al., 2004b). If this is the case, a time-course analysis would determine how long it takes to eliminate AM completely from the circulation of these mice. On the other hand, there may be a cross-reactivity of the kit's AM antibody with other members of the peptide family, such as AM2 or intermedin, which shares a 28% overall sequence identity with AM (Roh et al., 2004).

We observed that the diastolic blood pressure in the KOs was lower than in the WT animals. This seems to run against the generalized idea which identifies AM as a vasodilator (Lopez and Martinez, 2002). Nevertheless, this issue is more complex than initially perceived. Although AM is a vasodilator when injected peripherally (Santiago et al., 1995), it acts as a vasoconstrictor when injected into the brain, probably acting through vascular nerve terminals (Takahashi et al., 1994). Therefore, in a full-body knockout, the final outcome would depend on which physiological fraction, peripheral, or central, plays a more relevant role on blood pressure regulation. Similar results to the ones obtained in our model have been described when RAMP2, one of the components of the AM receptor, is deleted from endothelial cells (Koyama et al., 2013). So, it appears that the AM-regulated central control on vasodilatation is more powerful than the peripheral one.

The rapid weight gains and losses observed in our KO mice were unexpected. Careful observation of the animals, at the time they reached the peak of their weight gain, confirmed that this was due to generalized edema, which in some cases could be classified as anasarca. This is in line with the hydrops fetalis, a fetal generalized edema, which is described in all embryo knockout models of this gene and its receptors (Caron and Smithies, 2001; Shindo et al., 2001; Dackor et al., 2006). Interestingly, all fetuses die *in utero* due to the dysregulation of the blood and lymphatic vessels caused by the lack of AM (Fritz-Six et al., 2008) but our animals were able to recover swiftly and even lose all the extra weight in a few days. Obviously we have to conclude that adult mice have in place some extra mechanisms to deal with edema and are more protected than fetuses. Fortunately, no edema formation was observed in the ovarectomized females treated with small molecule 16311 or in KO mice not suffering anasarca, suggesting that lymphatic vessel blockade occurs only during these episodes.

Previous publications had centered their efforts on the effects of AM on specific bone cell types *in vitro* (Cornish et al., 1997, 2003; Naot et al., 2001; Uzan et al., 2008) or in *ex vivo* organ cultures (Cornish et al., 1997; Siclari et al., 2014), thus looking at the local influences of AM. As it happens with many hormones, AM has autocrine, paracrine, and endocrine functions that may be rather different (Larrayoz et al., 2014). This seems to be a clear example where local autocrine/paracrine loops result in bone deposition whereas long-distance endocrine signaling has the reverse effect. As shown by our results, this differential endocrine behavior may be elicited through indirect mechanisms involving the regulation of CGRP, insulin, glycemia, and ghrelin levels, although additional mechanisms may exist. CGRP belongs to the same peptide family as AM and it also signals through CRL, although using a different RAMP isoform (McLatchie et al., 1998). Previous studies using a conditional AM knockout model showed an increase in CGRP expression in the dorsal root ganglia and spinal cord of animals lacking AM in the neurons (Fernandez et al., 2010). Here we have shown that this peptide is also upregulated in the inducible KO model. Since CGRP has been involved in the recruitment and maturation of bone mesenchymal stem cells (Liang et al., 2015), this may provide an indirect mechanism linking AM reduction and increase of bone mass.

As another possibility, AM has been shown to reduce insulin secretion whereas a blocking monoclonal antibody against AM was able to increase insulin release five-fold (Martinez et al., 1996). This in turn would modulate blood glucose levels, being lower in the KOs. Low glycemic levels induce ghrelin secretion in the stomach through sympathetic neurons and the release of norepinephrine (Goldstein et al., 2011). Then ghrelin favors bone formation either in a direct way acting on osteoblasts (Ma et al., 2015) or through the induction of growth hormone or IGF-1 (Fukushima et al., 2005; Deng et al., 2008; Tong et al., 2012; Delhanty et al., 2014a,b; Wee and Baldock, 2014).

Other publications seem to support our findings when properly interpreted. For instance a study used the peptide fragment AM_22−52_, trying to take advantage of its immunomodulatory action, and found that it prevented bone loss in a model of collagen-induced arthritis (Ah Kioon et al., 2012). As explained above, this fragment is an AM inhibitor and, as such, it has similar effects to our small molecule inhibitor. Another recent publication was looking at the effects of AM and its inhibitors on the growth of bone-affecting tumors. The authors found that the inhibitory small molecule 16311, the same we use in this paper, reduced tumor induced osteolysis in their model (Siclari et al., 2014).

The potential clinical correlation between AM levels and osteoporosis has not been extensively studied. A manuscript, written in Chinese, found a significant increase of AM levels in human subjects with primary osteoporosis (Lin et al., 2001). Although it is difficult to establish whether this increase in AM is the cause or an effect of osteoporosis, according to our hypothesis, elevated levels of AM may result in a further loss of bone density and AM inhibitors could be indicated. Recently, it was found that a particular single nucleotide polymorphism (SNP) close to the *Adm* gene was responsible for a significant reduction in the circulating levels of AM (Cheung et al., 2011). As a consequence, the carriers of the minor allele are strongly protected against cancer (Martinez-Herrero and Martinez, 2013). It would be interesting to test whether these individuals are also protected from developing osteoporosis.

We found that small molecule 16311 was able to prevent bone loss in a mouse model of osteoporosis without eliciting any measurable toxicity, making this molecule a good candidate for a first-in-class drug against osteoporosis. Since AM is a vasodilator, most efficient inhibitors of this molecule should increase blood pressure, and this has been shown for 16311 (Martinez et al., 2004a). For the potential clinical development of this compound, this feature may not be too desirable. Interestingly, among the small molecules that were initially identified by their ability to bind AM and to reduce AM-induced cAMP elevation, there were some that did not increase blood pressure when injected in rats (Martinez et al., 2004a). These included small molecules 28086 and 37133. If these molecules are shown to possess an osteoporosis prevention effect they may be even more interesting than 16311 for further development. In the same line of thought, any inhibitor of AM could offer benefits to patients with diseases characterized by losses in bone mass. These could include monoclonal and polyclonal antibodies (Martinez et al., 1996; Ouafik et al., 2002; Kaafarani et al., 2009), inhibitory peptide fragments such as AM_22−52_ (Ishikawa et al., 2003), or small interfering RNA constructs (Ramachandran et al., 2007), among others. Nevertheless, small molecules continue to capture pharmacological attention due to their longer half-life and higher stability than peptides and antibodies.

## Author contributions

SM, IL, LO, LF, AA, and IO performed experiments and collected data. AM designed the study and experiments, and wrote the manuscript. All authors revised the manuscript and gave final approval to the latest version. All authors agree to be accountable for all aspects of the work.

## Funding

This study was supported by a grant from the Instituto de Salud Carlos III (PI13/02166), and FEDER.

### Conflict of interest statement

The authors declare that the research was conducted in the absence of any commercial or financial relationships that could be construed as a potential conflict of interest.
